# A Mixed-Field Circular and Non-Circular Source Localization Algorithm Based on Exact Spatial Propagation Geometry

**DOI:** 10.3390/s23146516

**Published:** 2023-07-19

**Authors:** Wei Lin, Weijia Cui, Bin Ba, Haiyun Xu, Jingjing Li

**Affiliations:** Institute of Information Engineering, PLA Strategic Support Force Information Engineering University, Zhengzhou 450001, China; linwei20221001@126.com (W.L.); xuhaiyun1995@163.com (H.X.); jing_57@163.com (J.L.)

**Keywords:** source localization, mixed field, circular and non-circular, exact spatial propagation geometry

## Abstract

In passive localization techniques, as the scale of the array of the sensors used increases, the source distribution may be a coexistence of near-field (NF) and far-field (FF) sources. Most of the existing algorithms dedicated to the localization of mixed-field sources are based on a simplified model, which has model errors and cannot make good use of non-circular properties when non-circular signals are present in the sources. In this paper, we present a mixed-field circular and non-circular source localization algorithm based on exact spatial propagation geometry. First, we make an initial estimate of the source parameters using exact spatial geometry relations. The MUSIC algorithm is then used in combination with the non-circular properties of the signal to achieve an accurate estimate. The algorithm does not lose performance due to model mismatch and is able to make good use of the non-circular properties of the sources to improve the estimation accuracy. The simulation results show that the proposed algorithm can effectively distinguish between sources and that the algorithm performs satisfactorily.

## 1. Introduction

Source localization is an important part of research in many areas, such as sonar, radar, mobile communications and wireless reconnaissance [1,2,3,4,5]. Range and direction of arrival (DOA) are the two main parameters in source localization, and the main existing high-resolution algorithms for performing DOA and range estimation are the multiple signal classification (MUSIC) [6] algorithm and the estimation of signal parameters via rotational invariance techniques (ESPRIT) [7] algorithm. With the evolution of communication technology, especially fifth-generation (5G) and sixth-generation (6G) mobile communication, massive and extremely large-scale, multiple-input multiple-output (MIMO) systems are gaining more and more attention. As the scale of antenna arrays enlarges and the signal frequency increases, the source distribution gradually becomes a coexistence of FF and NF sources. In this mixed-field source localization issue, the performance of localization algorithms tailored to FF or NF sources alone is generally unsatisfactory.

In recent years, researchers have also developed a number of algorithms that focus on the localization of mixed-field sources. In [8], Liang et al. proposed a two-stage MUSIC algorithm using cumulants for solving the mixed-field localization problem that avoids parameter matching and two-dimensional (2D) search. In [9], He et al. proposed a localization algorithm based on second-order statistics that separates FF sources from NF sources using an oblique projection technique and is more computationally efficient. In [10], Zheng et al. proposed an algorithm based on cumulant matrix reconstruction, which is less computationally complex and provides a more reasonable classification of sources. Ref. [11] used sparse arrays in mixed-field localization research, proposed a symmetric nested array (SNA) by symmetrizing the classical nested array and used a new mixed-order MUSIC algorithm, which improved resolution and accuracy. Sparse arrays have various advantages, such as large array aperture and low mutual coupling, and researchers have developed a multitude of sparse arrays for mixed-field source localization, including symmetrical double-nested array (SDNA) [12], improved symmetric nested array (ISNA) [13], symmetric displaced coprime array (SDCA) [14], symmetric flipped nested array (SFNA) [15], etc. Similarly, algorithms for sparse arrays have attracted the attention of a wider range of researchers. Shen et al. implemented mixed-field localization using subspace and sparse reconstruction techniques, which allowed the algorithm to exploit more unique lags and improved the accuracy of the algorithm [13]. Wu proposed a mixed sparse algorithm (MSA) [16] that utilizes both atomic norm and $l_{1}$-norm minimization algorithms and is applicable to generalized symmetric array configurations. A.M. Molaei et al. proposed a one-step algorithm [17] that utilizes the ESPIRT algorithm, eliminates the need for spectral peak search and thus greatly improves computational efficiency.

The above algorithms are based on simplified spatial models [18] that are approximated by second-order Taylor series expansion, so model errors are unavoidable. In addition, the elimination of parameters in the simplified model is usually conducted to avoid 2D search, which does not utilize all the data in the cumulant matrix and thus increases the estimation error. To avoid the problems associated with simplified models, Ref. [19] proposed an algorithm on the basis of exact spatial propagation geometry that avoids the performance losses associated with model mismatch. Refs. [20,21] built on this model to solve the array gain–phase error problem, and the cocentered orthogonal loop and dipole (COLD) array localization problem, respectively.

In this paper, on the basis of the exact spatial propagation geometry model, the circular and non-circular characteristics of sources are taken into account to achieve effective distinction and precise localization of the sources. Although some algorithms have started to investigate the problem of non-circular mixed-field source localization [22,23], these algorithms can only be applied to strictly non-circular signals and require multiple rank reduction processes.

By combining the exact spatial propagation geometry model and the characteristics of circular and non-circular signals, we propose a mixed-field circular and non-circular source localization algorithm on the basis of exact spatial propagation geometry. The proposed algorithm can effectively differentiate between source types and can take advantage of the non-circular properties of the signal to improve the estimation precision. First, we use array acceptance data to perform a fourth-order cumulant calculation to obtain the array manifold matrix. The initial estimation of the source parameters is then completed by constructing a system of equations based on the exact spatial propagation geometry of the source in relation to the array manifold matrix. Finally, on the basis of the initial estimation, the non-circular characteristic of the non-circular signal is used to differentiate between circular and non-circular signals and to complete the final estimation. Our major contributions can be summarized as follows:

(1) Using an exact spatial propagation geometry model combined with the characteristics of the source, we propose an algorithm to achieve effective differentiation and precise localization of mixed-field sources under the coexistence of circular and non-circular signals.
(2) We use a two-step estimation process by first solving the initial estimates using the exact space propagation geometry model and then conducting a local search using the classical MUSIC algorithm, which ensures estimation accuracy while reducing complexity.
(3) We adopt an alternating search method to solve for the valuation of the DOA and range parameters and introduce the non-circular properties of the signal to improve the performance of the algorithm.
(4) Various experimental simulations verified the superiority of the proposed algorithm.

The remaining sections of this paper are organized as follows: In Section 2, the signal model of mixed NF and FF sources is introduced. In Section 3, we propose a mixed NF and FF source localization algorithm on the basis of exact spatial propagation geometry for the coexistence of circular and non-circular signals. Performance simulations of the algorithm are reported in Section 4, and the full paper is summarized in Section 5.

*Notations:* Vectors (matrices) are denoted by bold-faced lower-case (upper-case) characters. The transpose, complex conjugate and conjugate transpose are denoted by ${\left(\cdot \right)}^{T}$, ${\left(\cdot \right)}^{*}$ and ${\left(\cdot \right)}^{H}$. $E\left(\cdot \right)$ represents the statistical expectation and $\left|\cdot \right|$ is the absolute value. The element in row *i* and column *j* of matrix A is represented by ${\langle A\rangle }_{i,j}$. ⊙ and ⊗ denote the Khatri-Rao product and Kronecker product. $\det\left\{A\right\}$ is the determinant of square matrix A.

## 2. The Signal Model

**Figure 1:**
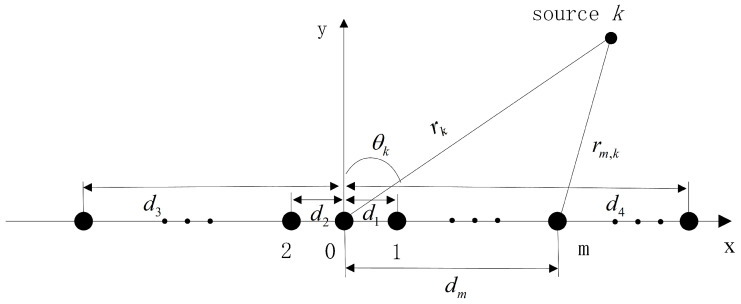
Linear array configuration.

Assume that the linear array consists of M+1 sensors, with sensor 0 located at the Cartesian origin as the reference sensor. There are *K* narrowband mixed FF and NF sources that impinge onto the array, of which the first $K_{1}$ sources are NF sources and the remaining $K-K_{1}$ sources are FF sources. The range from the *k*th source to the reference sensor is $r_{k}$ and the range to the *m*th sensor is $r_{m,k}$. The angle of incidence from the *k*th source to the array is $\theta_{k}$,which is the DOA of that source. The array model is shown in Figure 1. Based on the geometric relationships, the following equations can be constructed for $r_{k}$, $r_{m,k}$ and $\theta_{k}$:

(1) $$r_{m,k}=\sqrt{r_{k}^{2}+d_{m}^{2}-2d_{m}r_{k}\sin\theta_{k}},$$

where $d_{m}$ is the range between the *m*th sensor to the reference sensor.

The phase difference due to the different distances of the *k*th source to the reference array element and the *m*th array sensor is denoted as:

(2) $$a_{m}\left(\theta_{k},r_{k}\right)=e^{-j\tau_{m,k}}=e^{-j\frac{2\pi }{\lambda }\left(r_{k}-r_{m,k}\right)},$$

where $\tau_{m,k}$ denotes the time delay.

By arranging the phase changes due to time delays for the entire M+1 array elements as a column vector we get:

(3) $$a\left(\theta_{k},r_{k}\right)={\left[a_{1}\left(\theta_{k},r_{k}\right),a_{2}\left(\theta_{k},r_{k}\right),\ldots ,a_{M+1}\left(\theta_{k},r_{k}\right)\right]}^{T}.$$

The data from *K* sources received by the *m*th sensor of the array at moment *t* can be written as:

(4) $$x_{m}\left(t\right)=\sum_{k=1}^{K}s_{k}\left(t\right)a_{m}\left(\theta_{k},r_{k}\right)+n_{m}\left(t\right),$$

where $s_{k}\left(t\right)$ is the *k*th source signal and $n_{m}\left(t\right)$ denote the additive Gaussian white noise. Equation (4) can be expressed in matrix form as:

(5) $$x\left(t\right)=\sum_{k=1}^{K}a\left(\theta_{k},r_{k}\right)s_{k}\left(t\right)+n\left(t\right)=\mathrm{As}\left(t\right)+n\left(t\right),$$

where $s\left(t\right)={\left[s_{1}\left(t\right),s_{2}\left(t\right),\ldots ,s_{K}\left(t\right)\right]}^{T}$ is the *K* narrowband sources, $A=[a\left(\theta_{1},r_{1}\right),\ldots ,a\left(\theta_{K},r_{K}\right)]$ is the array manifold matrix and n(t) denotes the noise vector.

Now assume that the *K* sources contain $K_{c}$ circular signals and $K_{nc}$ non-circular signals. The covariance matrix of the receiving signal x(t) is expressed as:

(6) $$R=E\left\{x\left(t\right)x^{H}\left(t\right)\right\}=AR_{ss}A+\sigma_{n}^{2}I_{N},$$

where $R_{ss}=E\left\{s\left(t\right)s^{H}\left(t\right)\right\}$. And the unconjugated covariance matrix of x(t) can be denoted as:

(7) $$\begin{array}{cc} {R^{\prime }}_{xx} & =E\left\{x\left(t\right)x^{T}\left(t\right)\right\}=A_{nc}{R^{\prime }}_{nc}A_{{}_{nc}}^{T}\\ \; & =\sum_{k=1}^{K_{nc}}\rho_{k}e^{j\varphi_{k}}\sigma_{k}^{2}a\left(\theta_{k,nc},r_{k,nc}\right)a^{H}\left(\theta_{k,nc},r_{k,nc}\right), \end{array}$$

where ${R^{\prime }}_{nc}=E\left\{s_{nc}\left(t\right)s_{nc}^{T}\left(t\right)\right\}$ is the unconjugated covariance matrix of non-circle signal and $A_{nc}=\left[a\left(\theta_{nc,1},r_{nc,1}\right),\ldots ,a\left(\theta_{nc,K_{nc}},r_{nc,K_{nc}}\right)\right]$ is the steering matrix. For the *k*th non-circular signal, $\rho_{k}$ represents the non-circular rate and $\varphi_{k}$ is the non-circular phase. When the source is a circular signal, the value of ${R^{\prime }}_{xx}$ is zero; while when the source is a non-circular signal, ${R^{\prime }}_{xx}$ is a non-zero value. Because of this, non-circular signals can be estimated with increased accuracy using unconjugated covariance matrix to increase the dimensionality of the matrix during the MUSIC spectral peak search.

**Remark** **1.** *Models* (1) *and* (2) *are applicable to both FF and NF sources. For NF sources, the model is able to solve well for both DOA and range parameters. However, for FF sources, the angle parameter dominates the data received by the array, with variations in range having a minimal effect on the data received by the array. Therefore, the FF source can also be used to solve for the angular parameters using the model, but with a larger error in the estimation of range. The simulations show that when the above model are used for parameter estimation of FF sources, the model error has a large effect on the range and an accurate distance valuation cannot be obtained, but it has a small effect on the DOA valuation and a relatively accurate DOA valuation can be obtained. Fortunately, for FF sources, in practice we are also only concerned with DOA valuation.*

## 3. Algorithm Used

We propose an algorithm for the localization of mixed-field circular and non-circular signals based on an exact model. The algorithm can be divided into the following steps: (1) solving for the array manifold matrix, (2) initial estimation of the DOA and range of the mixed-field sources and (3) final estimation of the circular and non-circular signals.

### 3.1. Solving for the Array Manifold Matrix

The the array manifold matrix A contains all the information about the DOA and range of the target source, which can be used wisely to solve for the DOA and range estimates of the source. In this paper, the array manifold matrix A is solved based on the array acceptance data using low-rank decomposition method.

The algorithm requires the use of the fourth-order cumulants of x(t), which is defined as follows [24]:

(8) $$\begin{array}{cc} cum\left(x_{m},x_{n}^{*},x_{p},x_{q}^{*}\right) & =E\left\{x_{m}x_{n}^{*}x_{p}x_{q}^{*}\right\}\\ \; & -\;E\left\{x_{m}x_{p}\right\}E\left\{x_{n}^{*}x_{q}^{*}\right\}\\ \; & -\;E\left\{x_{m}x_{n}^{*}\right\}E\left\{x_{p}x_{q}^{*}\right\}\\ \; & -\;E\left\{x_{m}x_{q}^{*}\right\}E\left\{x_{n}^{*}x_{p}\right\}, \end{array}$$

where $x_{m}$ represents the *m*th element of the vector x. Based on the definition of the fourth-order cumulants in Equation (8), the following cumulative volume matrix is constructed:

(9) $$Z_{i}=cum(x_{i}\left(t\right),x_{0}^{*}\left(t\right),x\left(t\right),x^{H}\left(t\right))=A\Phi_{i}\eta A^{H},i=0,1,2,\ldots ,M.$$

where $\eta =cum\left(s_{i}\left(t\right),s_{i}^{*}\left(t\right),s_{i}\left(t\right),s_{i}^{*}\left(t\right)\right)$ is the signal kurtosis and $\Phi_{i}=diag\left[e^{j\tau_{i,1}},\ldots ,e^{j\tau_{i,M}}\right]$.

Letting i=0,1,2,…,M yields a number of M+1 matrices, which are used to construct a new matrix Z in the following way:

(10) $$Z={\left[Z_{0}^{T},Z_{1}^{T},\ldots ,Z_{M}^{T}\right]}^{T}.$$

The matrix Z in Equation (10) can be represented by a matrix of signal kurtosis and array manifold matrix, specifically [19]:

(11) $$Z=\eta \left(A⊙A\right)A^{H},$$

The low-rank decomposition of the matrix Z yields the array manifold matrix A accordingly [21,25]. From matrix A we can calculate the phase difference of the *k*th source between the reference sensor and the *m*th sensor:

(12) $${\hat{a}}_{m}\left(\theta_{k},r_{k}\right)={\langle A\rangle }_{m,k}=e^{-j{\hat{\varphi }}_{m,k}}.$$

### 3.2. Initial Estimation of the DOA and Range of the Mixed-Field Sources

Equation (2) expresses the phase delay relationship through an exact geometric relationship, and Equation (12) determines the phase delay using the acceptance data, which are equivalent, and further we can obtain:

(13) $$\frac{2\pi }{\lambda }\left(r_{k}-r_{m,k}\right)={\hat{\varphi }}_{m,k}.$$

Letting m = 1 and 2, the following system of equations can be created:

(14) $$\left\{\begin{array}{c} \frac{2\pi }{\lambda }\left(r_{k}-\sqrt{r_{k}^{2}+d_{1}^{2}-2d_{1}r_{k}\sin\theta_{k}}\right)={\hat{\varphi }}_{1,k}\\ \frac{2\pi }{\lambda }\left(r_{k}-\sqrt{r_{k}^{2}+d_{2}^{2}-2d_{2}r_{k}\sin\theta_{k}}\right)={\hat{\varphi }}_{2,k} \end{array}.\right.$$

The two unknown parameters $r_{k}$ and $\theta_{k}$ can be estimated as:

(15) r^kuni=φ^1,k2π2πλλ2d2−φ^2,k2π2πλλ2d1−d12d2+d1d222φ^1,k2π2πλλd2−2φ^2,k2π2πλλd1,

(16) θ^kuni=arcsin2r^kuniφ^1,k2π2πλλ−φ^1,k2π2πλλ2+d122r^kunid1.

To avoid phase ambiguities, the positions of $d_{1}$ and $d_{2}$ need to be restricted, specifically, $d_{1}\leq \lambda /2$ and d2 $d_{2}\leq \lambda /2$. According to Equations (15) and (16) we have been able to achieve estimates of the parameters $r_{k}$ and $\theta_{k}$, but the estimation errors are relatively large due to the limitations of $d_{1}$ and $d_{2}$.

To improve the estimation accuracy, we selected the two sensors furthest away from the reference sensor and reconstructed the set of equations according to Equation (13). As the distance between sensors may exceed λ/2, the spatial phase may likewise be ambiguous. The new set of equations can be expressed as

(17) $$\left\{\begin{array}{c} \frac{2\pi }{\lambda }\left(r_{k}-\sqrt{r_{k}^{2}+d_{3}^{2}-2d_{3}r_{k}\sin\theta_{k}}\right)={\hat{\varphi }}_{3,k}+2n_{3}\pi \\ \frac{2\pi }{\lambda }\left(r_{k}-\sqrt{r_{k}^{2}+d_{4}^{2}-2d_{4}r_{k}\sin\theta_{k}}\right)={\hat{\varphi }}_{4,k}+2n_{4}\pi \end{array},\right.$$

where $d_{3}$ and $d_{4}$ are the two sensors furthest away from the reference sensor. $n_{3}$ and $n_{4}$ are whole-cycle ambiguities in phase due to excessive distances. Solving the above system of equations yields:

(18) r^kmul=φ^3,k+2n3π2π2πλλ2d4−φ^4,k+2n4π2π2πλλ2d3−d32d4+d3d422φ^3,k+2n3π2π2πλλd4−2φ^4,k+2n4π2π2πλλd3,

(19) θ^kmul=arcsin2r^kmulφ^3,k+2n3π2π2πλλ−φ^3,k+2n3π2π2πλλ2+d322r^kmuld3.

Because the exact values of $n_{3}$ and $n_{4}$ cannot be determined, there may be multiple solutions to Equations (18) and (19). Fortunately, we can rule out ambiguous solutions by the solutions of Equations (15) and (16), i.e., finding the closest value to Equations (15) and (16) in the fuzzy solution of Equations (18) and (19) which is the initial estimate of the angle and range.

(20) $$\left({\hat{r}}_{k}^{ini},{\hat{\theta }}_{k}^{ini}\right)=\underset{{\hat{r}}_{k,i}^{mul},{\hat{\theta }}_{k,i}^{mul}}{\arg\min}\left\{\left|{\hat{r}}_{k,i}^{mul}-{\hat{r}}_{k}^{uni}\right|+\left|{\hat{\theta }}_{k,i}^{mul}-{\hat{\theta }}_{k}^{uni}\right|\right\},$$

where $r_{k,i}^{mul}$ and $\theta_{k,i}^{mul}$ represent the *i*th solution of the multiple ambiguous solutions in Equations (18) and (19).

### 3.3. Final Estimate of DOA and Range of Circle and Noncircle Mixed-Field Sources

From initial estimates, we have obtained a rough estimate of the DOA and range of the mixed-field sources and are generic for circular and non-circular signals. The above solution procedure only makes use of the geometric relationship of the array and the estimation accuracy is limited. The classical MUSIC algorithm achieves high accuracy DOA estimation based on the orthogonality of the noise and signal subspaces, on which the non-circular properties of the sources can be exploited to further improve the estimation precision and also to distinguish between circular and non-circular signals.

First, the distinction between circular and non-circular sources can be achieved by using the circular signal with zero unconjugated covariance and the non-circular signal with non-zero unconjugated covariance. The eigenvalue decomposition of the unconjugated covariance matrix ${R^{\prime }}_{xx}$ yields the noise subspace and signal subspace:

(21) $${R^{\prime }}_{xx}=Q_{sc}\Lambda_{sc}Q_{sc}+Q_{nc}\Lambda_{nc}Q_{nc},$$

where $\Lambda_{sc}$ and $\Lambda_{nc}$ correspond to large and small eigenvalues of ${R^{\prime }}_{xx}$, $Q_{sc}$ and $Q_{nc}$ are the signal subspace and the noise subspace respectively. Non-circular sources can be distinguished by the two-dimensional spectral peak search function as follows:

(22) $$f_{nc}\left(r,\theta \right)=\frac{1}{a^{H}\left(\theta ,r\right)Q_{nc}Q_{nc}^{H}a\left(\theta ,r\right)}.$$

To reduce the computational complexity of the two-dimensional search, the search can be carried out in the immediate region of the initial estimates ${\hat{r}}_{k}^{ini}$ and ${\hat{\theta }}_{k}^{ini}$.

Next, we use an alternating search method to achieve the final estimates of the range and angle of the mixed-field circular and non-circular sources.   

To exploit the non-circular property, we construct new data vectors using the array acceptance data and their conjugate vectors:

(23) $$y\left(t\right)=\left[\begin{array}{c} x\left(t\right)\\ x^{*}\left(t\right) \end{array}\right]=\left[\begin{array}{c} A\\ A^{*}\Psi^{*} \end{array}\right]s\left(t\right)+\left[\begin{array}{c} n\left(t\right)\\ n^{*}\left(t\right) \end{array}\right],$$

where $\Psi =diag{\left\{e^{j\psi_{k}}\right\}}_{k=1}^{K_{nc}}$, and $\psi_{k}$ is the noncircular phase. The covatiance matrix of $y\left(t\right)$ can be expressed as:

(24) $$R_{y}=E\left[y\left(t\right)y^{H}\left(t\right)\right]=\left[\begin{array}{cc} R & {R^{\prime }}_{xx}\\ {R^{\prime }}_{xx}^{*} & R^{*} \end{array}\right].$$

Performing eigendecomposition of $R_{y}$ we can obtain:

(25) $$R_{y}=U_{ys}\Lambda_{ys}U_{ys}^{H}+U_{yn}\Lambda_{yn}U_{yn}^{H},$$

where $\Lambda_{ys}$ and $\Lambda_{yn}$ are eigenvalues, $U_{ys}$ and $U_{yn}$ are the signal subspace and the noise subspace.

Due to the orthogonality of the array manifold vector to the noise subspace, we can obtain:

(26) $$\begin{array}{c} U_{yn}^{H}a\left(\theta_{k},r_{k},\psi_{k}\right)=U_{yn}^{H}\left[\begin{array}{c} a\left(\theta_{k},r_{k}\right)\\ a^{*}\left(\theta_{k},r_{k}\right)e^{j\psi_{k}} \end{array}\right]\\ =U_{yn}^{H}\left[\begin{array}{cc} a\left(\theta_{k},r_{k}\right) & \\ & a^{*}\left(\theta_{k},r_{k}\right) \end{array}\right]\left[\begin{array}{c} 1\\ e^{j\psi_{k}} \end{array}\right]=0. \end{array}$$

According to the rank reduction (RARE) principle [23,26], we can obtain

(27) $$U_{yn}^{H}\left[\begin{array}{cc} a\left(\theta_{k},r_{k}\right) & \\ & a^{*}\left(\theta_{k},r_{k}\right) \end{array}\right]=0$$

Due to the influence of errors such as noise and finite number of snapshots, the matrix $U_{yn}^{H}$ and the matrix $\left[\begin{array}{cc} a\left(\theta_{k},r_{k}\right) & \\ & a^{*}\left(\theta_{k},r_{k}\right) \end{array}\right]$ cannot be completely orthogonal in practice. Therefore, the spectral peak search function $f\left(\theta ,r\right)$ is defined, and $f\left(\theta ,r\right)$ achieves its maximum value only when $\theta =\theta_{k}$ and $r=r_{k}$:

(28) $$f\left(\theta ,r\right)=\frac{1}{\det\left\{Q\left(\theta ,r\right)\right\}}=\frac{1}{\det\left\{V^{H}\left(\theta ,r\right)U_{yn}U_{yn}^{H}V\left(\theta ,r\right)\right\}},$$

where $V\left(\theta ,r\right)=\left[\begin{array}{cc} a\left(\theta ,r\right) & \\ & a^{*}\left(\theta ,r\right) \end{array}\right]$.

The computational complexity of a direct two-dimensional spectral peak search is high, and we use an alternating search method to reduce the complexity while improving the estimation accuracy in the region close to the initial estimate.

The valuation of the DOA $\theta_{k}$ and the range $r_{k}$ can be obtained by searching alternately by the following equations:

(29) $${\hat{\theta }}_{k}^{fin}=\underset{\theta }{\arg\max}\left\{\frac{1}{\det\left\{V^{H}\left(\theta ,\hat{r}\right)U_{yn}U_{yn}^{H}V\left(\theta ,\hat{r}\right)\right\}}\right\},$$

(30) $${\hat{r}}_{k}^{fin}=\underset{r}{\arg\max}\left\{\frac{1}{\det\left\{V^{H}\left(\hat{\theta },r\right)U_{yn}U_{yn}^{H}V\left(\hat{\theta },r\right)\right\}}\right\}.$$

The initial values calculated for the above iterations can be obtained from Equation (20). The details of this alternating iteration algorithm are summarised in Algorithm 1.
**Algorithm 1:** Alternating iteration algorithm1: **Initialisation:** Maximum number of iterations N=50, iteration error $\varepsilon =10^{-3}$, Upper boundary of the Fresnel region $r_{F}$.2: **Results:** DOA and range estimation $({\hat{\theta }}_{k}^{fin},{\hat{r}}_{k}^{fin}).$3: Calculate the covariance matrix $R_{y}$ (24) and obtain the noise subspace $U_{yn}$ (25).4: **for** k=1:K5:    Set i=1, obtain initial ${\hat{\theta }}_{k}\left(0\right)={\hat{\theta }}_{k}^{ini}$ and ${\hat{r}}_{k}\left(0\right)={\hat{r}}_{k}^{ini}$ from (20).6:    **Do**7:       **If:** ${\hat{r}}_{k}\left(0\right)\leq r_{F}$8:          ${\hat{r}}_{k}\left(i\right)=\underset{r}{\arg\max}\frac{1}{\det\left\{V^{H}\left({\hat{\theta }}_{k}\left(i-1\right),r\right)U_{yn}U_{yn}^{H}V\left({\hat{\theta }}_{k}\left(i-1\right),r\right)\right\}}$9:          ${\hat{\theta }}_{k}\left(i\right)=\underset{\theta }{\arg\max}\frac{1}{\det\left\{V^{H}\left(\theta ,{\hat{r}}_{k}\left(i\right)\right)U_{yn}U_{yn}^{H}V\left(\theta ,{\hat{r}}_{k}\left(i\right)\right)\right\}}$10:       **Else:**11:          ${\hat{r}}_{k}\left(i\right)=\infty$12:          ${\hat{\theta }}_{k}\left(i\right)=\underset{\theta }{\arg\max}\frac{1}{\det\left\{V^{H}\left(\theta ,{\hat{r}}_{k}\left(i\right)\right)U_{yn}U_{yn}^{H}V\left(\theta ,{\hat{r}}_{k}\left(i\right)\right)\right\}}$13:       **End if:**14:       i=i+1.15:   **While** i≤N and $\left|{\hat{\theta }}_{k}\left(i\right)-{\hat{\theta }}_{k}\left(i-1\right)\right|\geq \varepsilon$.16:    ${\hat{r}}_{k}^{fin}={\hat{r}}_{k}\left(i\right)$, ${\hat{\theta }}_{k}^{fin}={\hat{\theta }}_{k}\left(i\right)$.17: **end for**

**Remark** **2.** *Equation* (22) *can estimate non-circular sources only, and Equation* (28) *can estimate both circular and non-circular sources. Note that Equation* (22) *only utilises the unconjugated covariance matrix, while Equation* (28) *makes full use of the covariance matrix and the unconjugated covariance matrix. Therefore, Equation* (28) *is more accurate and Equation* (22) *is used to differentiate between circular and non-circular signals.*

## 4. Simulation Results

In this section, the performance of the proposed algorithm for the localization of mixed-field sources in the presence of co-existing circular and noncircular signals is verified through several sets of simulation results. We compare the proposed algorithm with the Mixed-order MUSIC [11] algorithm, the Mixed Sparse Approach (MSA) algorithm [16], the fine-estimation (Fine) and refine-estimation (MILE) in the exact model [19] algorithm, and the Cramér-Rao bound (CRB) [9]. For comparison with other algorithms, we have used symmetric formations in this section of simulations and the minimum inter-sensor spacing is set to d=λ/4. The parameter β in the MSA algorithm adopts 0.5. The source signals are all mutually independent non-Gaussian random processes and the noise is additive Gaussian white noise.

We evaluated the performance of the algorithm by means of root mean square error (RMSE) from a large number of Monte Carlo experiments:

(31) $$\mathrm{RMSE}=\sqrt{\frac{1}{\kappa \eta }\sum_{i=1}^{\eta }\sum_{k=1}^{\kappa }{\left(\alpha_{k}^{i}-\alpha_{k}\right)}^{2}},$$

where η=500 is the number of Monte Carlo experiments. $\alpha_{k}$ is the real $r_{k}$ or $\theta_{k}$, $\alpha_{k}^{i}$ denotes the estimated $r_{k}$ or $\theta_{k}$ for the *i*th experiment, correspondingly. κ represents the number of different types of sources, e.g., to obtain the RMSE curve for the NF circular sources, κ is the number of NF circular sources.

### 4.1. Sources Distinction

**Figure 2:**
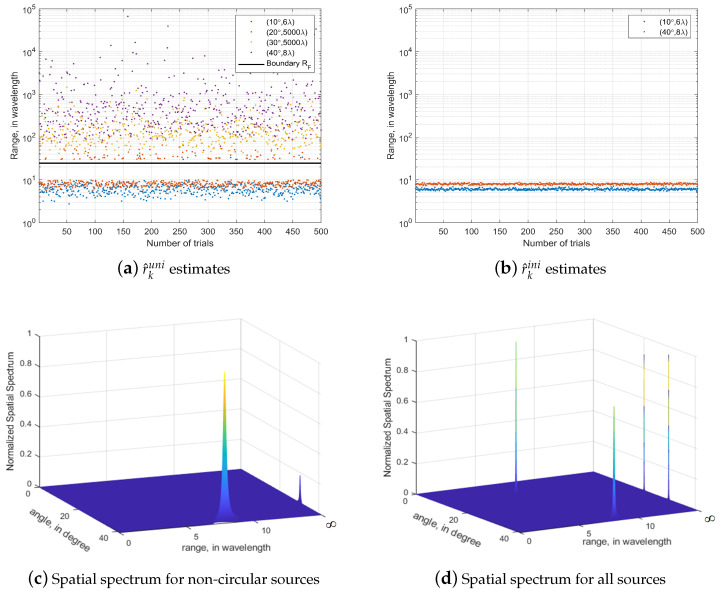
Sources distinction for mixed-field circular and noncircular sources. (SNR = 20 dB and T = 10,000).

In the first simulation, we verify the source distinction capability of the proposed algorithm. We considered four narrowband sources impinge onto a ULA with the sensors number of 15. The sources are two circular signals $\left(10^{\circ },6\lambda \right)$, $\left(20^{\circ },5000\lambda \right)$ and two non-circular signals $\left(30^{\circ },5000\lambda \right)$, $\left(40^{\circ },8\lambda \right)$ respectively. On the premise of the initial estimation of Equation (20), we simulated the normalised spectrum of the source using Equations (22) and (28), and the simulation results are shown in Figure 2.

The distinction between NF and FF sources is mainly based on the estimation of the range of the source. When the range valuation lies in the Fresnel region, the source is determined to be an NF source, and when the range valuation is much larger than the upper boundary of the Fresnel region, the source is an FF source. Figure 2a shows the simulation results for ${\hat{r}}_{k}^{uni}$ in Equation (15) in the initial estimation, and Figure 2b shows the simulation results for ${\hat{r}}_{k}^{ini}$ in Equation (20). The simulation results show that, after the initial estimation, we have been able to effectively distinguish FF sources and NF sources. The normalised spectral peaks in Figure 2c are derived from Equation (22), which can be seen to estimate only non-circular signals; the normalised spectral peaks in Figure 2d are obtained from Equation (28), a search formula capable of estimating both circular and non-circular signals. From Figure 2, the proposed algorithm is able to distinguish between mixed-field sources where circular and non-circular signals coexist. As we have already made initial estimates of angle and range, the computational complexity is relatively low as the 2D spectral peak search only needs to be performed over a smaller area.

### 4.2. The ULA Case

**Figure 3:**
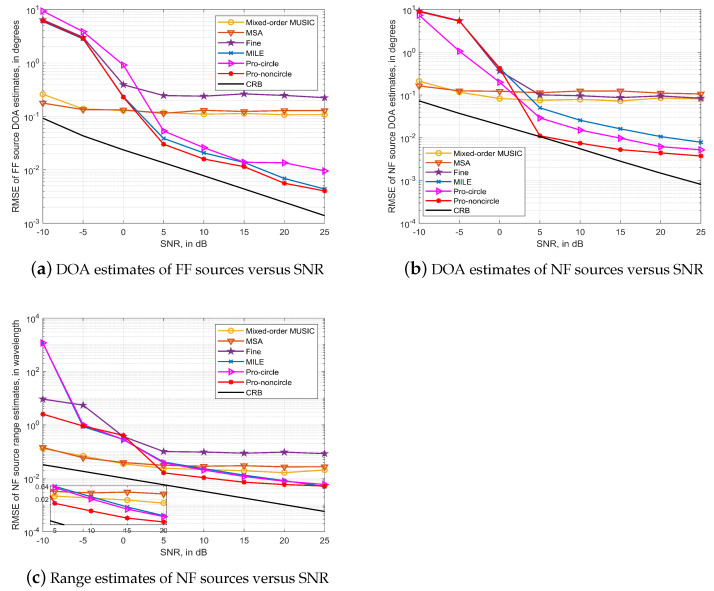
Algorithms performance in Symmetric ULA case with 15 sensors and *T* = 20,000.

In the second simulation, we simulate and verify the estimation performance of the algorithm in the symmetric ULA. Considering the same array configuration and sources distribution as in simulation A, the curves of RMSE variation with SNR for several algorithms are shown in Figure 3, where the number of snapshots is 20,000.

The simulation results show that the Mixed-order MUSIC algorithm and the MSA algorithm based on the simplified model have poor performance. The Fine algorithm, which relies only on an accurate spatial geometry model, also performs poorly under the assumed simulation conditions. The estimation performance of the proposed algorithm, on the other hand, is significantly better than several other algorithms, especially in the estimation of non-circular sources. This is because the proposed algorithm is able to exploit the non-circular property of the sources to obtain covariance matrices of greater dimensionality, thus improving the estimation accuracy.

In the case of low SNR, the ${\hat{\varphi }}_{m,k}$ value calculated by the exact spatial propagation geometry algorithm differs significantly from the true value, so that the error in the results obtained when solving the system of equations of Equation (14) is large, resulting in the final inability to obtain an accurate parameter valuation. In contrast, both Mixed-order MUSIA and MSA algorithms are based on the spectral peak search method, so they are superior to the proposed algorithm in low SNR.

### 4.3. The SLA Case

In this simulation, we assume the case of four circular and non-circular coexisting mixed-field sources impinging on a symmetric SLA. The four sources are two circular $\left(10^{\circ },6\lambda \right)$, $\left(20^{\circ },5000\lambda \right)$ and two non-circular $\left(30^{\circ },5000\lambda \right)$, $\left(40^{\circ },8\lambda \right)$ signals, the sensors are located at [−12,−9,−7,−5,−2,−1,0,1,2,5,7,9,12]d, and the number of snapshots is 20,000.

**Figure 4:**
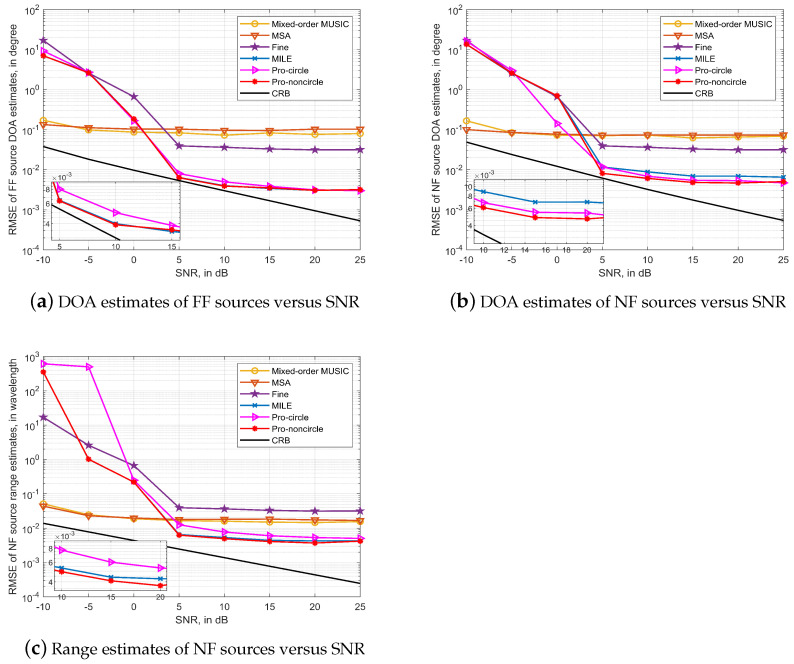
Algorithms performance in Symmetric SLA case with *T* = 20,000.

The Mixed-order MUSIC and MSA algorithms based on the simplified model have the worst algorithm performance in the SLA case, due to the fact that the Mixed-order MUSIC algorithm can only exploit continuous lags, while the MSA algorithm needs to fill the holes of the sparse array, processes that introduce errors and cause degradation in algorithm performance. In contrast, algorithms based on exact spatial geometry models have no data loss or other errors introduced, and therefore do not introduce performance degradation in the symmetric SLA case. Similarly, the proposed algorithm outperforms the MILE algorithm due to the exploitation of the non-circular property of the sources and, as shown in Figure 4b, the performance improvement in estimation of NF sources is more pronounced.

### 4.4. Impact of Snapshots on Algorithm Performance

**Figure 5:**
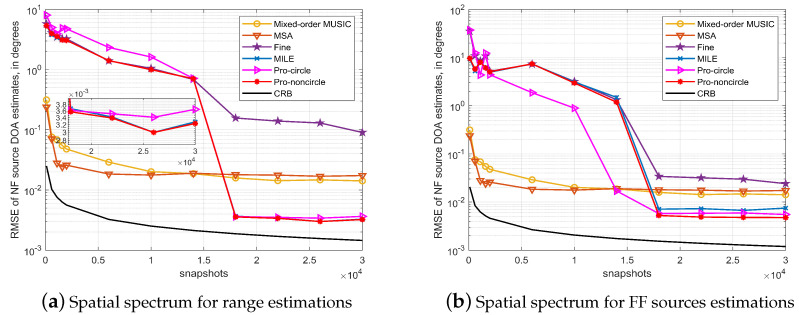
Impact of snapshots on algorithm performance. (SNR = 15 dB).

We simulate the effect of the number of snapshots on the proposed algorithm. The same array and source distribution as in simulation C is used, and the SNR is set to 15 dB. The simulation results are shown in Figure 5.

For a large number of snapshots, the algorithm outperforms several other algorithms significantly, while performance is poorer for a smaller number of snapshots. This is due to the fact that the proposed algorithm uses fourth-order cumulants to calculate the array manifold during the initial estimation, and only a sufficiently large number of snapshots can guarantee accurate results.

## 5. Conclusions

This paper presents a mixed-field circular and non-circular sources localization algorithm based on exact spatial propagation geometry, which makes full utilization of the spatial geometry model of the source, the classical MUSIC algorithm and the non-circular properties of the incident sources. A first initial solution is performed using an exact spatial propagation geometry model, and then the non-circular properties of the sources are combined with the MUSIC algorithm to solve the mixed-field parameter estimation problem using a local alternating search method. The simulation results show that the proposed algorithm can effectively distinguish the sources and has a better DOA and range estimation performance.

## Data Availability

The data that support the findings of this study are available upon request from the authors.
